# Macrophage Proangiogenic VEGF-A Is Required for Inflammatory Arteriogenesis During Vascular Injury

**DOI:** 10.3390/biomedicines13040828

**Published:** 2025-03-31

**Authors:** Sheila Sharma, Julia Pierce, Jade C. Neverson, Rachel Khan, Cadence F. Lee, Saketh Uppuluri, Crystal Parry, Elizabeth Amelotte, Celia A. Butler, Frank W. Sellke, Elizabeth O. Harrington, Gaurav Choudhary, Alan R. Morrison, Chris S. Mantsounga

**Affiliations:** 1 Vascular Research Laboratory, Providence VA Medical Center, Providence, RI 02908, USA; 2 Ocean State Research Institute, Inc., Providence, RI 02908, USA; 3 Department of Internal Medicine, Alpert Medical School of Brown University, Providence, RI 02903, USA; 4 Cardiovascular Research Center, Brown University Health, Rhode Island Hospital, Providence, RI 02903, USA

**Keywords:** angiogenesis, inflammation, VEGF-A, macrophage, vascular biology

## Abstract

**Background:** Peripheral artery disease is associated with significant morbidity and mortality. Mechanical revascularization strategies are a mainstay of treatment but are often limited by the anatomic complexity of atherosclerotic lesions. Therapeutic angiogenesis has fallen short of being impactful due to fundamental gaps in our understanding of postdevelopmental angiogenesis. **Methods:** Using a preclinical model of peripheral artery disease involving acute vascular injury by femoral artery ligation along with cellular and molecular studies of VEGF-A expression, we sought to further understand the early role of macrophages in inflammatory angiogenesis and arteriogenesis. **Results:** Macrophage depletion studies revealed that the optimal levels of tissue VEGF-A expression, endothelial cell recruitment, and blood flow recovery were dependent on early macrophage recruitment. Proangiogenic VEGF-A expression was highest in macrophages polarized towards an inflammatory phenotype. Myeloid VEGF-A-deletion, while having no impact on the potent inflammatory cytokine, IL-1β, led to reductions in ischemic tissue VEGF-A, endothelial cell recruitment, and blood flow recovery due to impaired angiogenesis and arteriogenesis. Transplant of inflammatory polarized macrophages rescued the myeloid VEGF-A-deletion phenotype, leading to full blood flow recovery. **Conclusions:** Macrophages are a necessary and sufficient source of tissue VEGF-A during inflammatory-driven angiogenesis and arteriogenesis in response to vascular injury. Although further study is needed, cell-based therapeutic angiogenesis strategies involving the polarization of macrophages toward an inflammatory state, in order to produce high levels of proangiogenic VEGF-A, may be quite effective for improving revascularization in the context of PAD.

## 1. Introduction

Peripheral artery disease (PAD), consequent to systemic atherosclerosis, can lead to tissue damage through both acute and chronic occlusive ischemia, resulting in significant morbidity and mortality [1,2,3]. It is estimated that over 230 million people worldwide suffer from PAD [4,5]. Critical limb ischemia (CLI) is the most severe manifestation of PAD, and without timely diagnosis and revascularization, patients with CLI are at increased risk of amputation and mortality [2]. Treatment options are often limited to mechanical revascularization, by surgical bypass or angioplasty, and while revascularization improves the rates of limb salvage, it is often incomplete due to the complexity of the atherosclerotic lesions and consequent vascular anatomy [2,6]. Failure to heal after revascularization is influenced by infection, neuropathy, and renal failure, and mortality in these patients can be as high as 50% at 5 years [7]. The concept of therapeutic angiogenesis as a strategy for revascularization has been around for decades; however, clinical trials utilizing relatively simple strategies involving single growth factors or signaling pathways have fallen short of impacting clinical outcomes [8]. This impaired translation to the clinic is likely due to an incomplete understanding of the molecular and multicellular orchestration of postdevelopmental angiogenesis and arteriogenesis, indicating a critical need to further define the complex mechanisms involved.

While maladaptive inflammation has been associated with worsening outcomes in patients with PAD [9,10], there has been increasing appreciation for the role of early inflammatory macrophages in orchestrating new vessel growth and maturation [11,12]. Macrophages appear to be a critical source of proangiogenic vascular endothelial growth factor A (VEGF-A) during acute ischemia or in wound injury models [12]. Early infiltrating macrophages contribute as much as 50–75% of the VEGF-A protein in ischemic muscle tissue within the first 3 days after femoral artery ligation [13,14]. In the setting of vascular injury, circulating monocytes from the bone marrow and spleen are recruited to the infarcted area, in part through CCL2-CCR2 signaling [15,16]. The early inflammatory monocytes are highly invasive, accumulating in the infarct zone and differentiating into macrophages to help clear dead cells and debris. These macrophages are largely polarized toward a “classic” inflammatory phenotype because of their association with the Th1 T cell cytokine environment [17]. In humans, the classic inflammatory monocyte/macrophage populations express high levels of the markers CC-motif chemokine receptor 2 (CCR2), lipopolysaccharide (LPS) co-receptor CD14, interleukin-1β (IL-1β), interleukin-6 (IL-6), and inducible nitric oxide synthase (iNOS) [12]. The reparative phase results from the resolution of the inflammatory phase and is typically characterized by phenotypic transition to the anti-inflammatory or “alternatively activated” macrophage phenotypes, which resemble resident tissue macrophages under gene expression profiling. Alternatively activated macrophages contribute to wound closure and are marked by high levels of the chemokine receptor CX3CR1 and the type III Fcγ receptor CD16. One characteristic of the proliferative and healing phase is the eventual downregulation of pro-inflammatory mediators and the upregulation of anti-inflammatory cytokines like transforming growth factor beta (TGF-β), interleukin-10 (IL-10), and IL-1 receptor antagonist (IL-1ra) [18,19,20].

Angiogenesis can also be regulated by the alternative splicing of VEGF-A, leading to an alternate isoform with opposing biological activity. The splicing event occurs in exon 8, leading to an anti-angiogenic VEGF-A_165_b isoform that differs in six amino acids [21]. Proangiogenic VEGF-A_165_a binds to VEGFR2, leading to traditional autophosphorylation, with effector signaling that promotes angiogenesis, whereas VEGF-A_165_b causes an altered VEGFR2 tyrosine phosphorylation pattern that consequently inhibits angiogenesis [22]. Macrophage expression of VEGF-A_165_b can inhibit revascularization of the ischemic hindlimbs, and the mechanism appears to occur via autocrine induction of an inflammatory phenotype that impairs angiogenesis through macrophage VEGFR1 [23]. However, some conflicting clinical data with regard to the upregulation of antiangiogenic VEGF-A_165_b have been collected; serum VEGF-A_165_b is elevated with peripheral arterial disease but reduced in patients with critical limb ischemia relative to those with intermittent claudication or normal blood flow [23,24,25]. We recently determined that transcription of the proangiogenic VEGF-A_165_a isoform, relative to VEGF-A_165_b, was critically dependent on macrophage IL-1β expression in inflammatory macrophages [14]. Moreover, macrophage IL-1β was absolutely required for postdevelopmental arteriogenesis in the hindlimb ischemia model, likely secondary to IL-1β-driven proangiogenic VEGF-A expression. A major role of IL-1β in inflammatory angiogenesis and arteriogenesis appears to be to ensure the VEGF-A-driven recruitment of endothelial cell precursors to the ischemic site [26]. However, while developmental vascularization depends on VEGF-A chemotactic gradients, prior studies have indicated that macrophage VEGF-A expression was dispensable relative to macrophage function promoting endothelial cell fusion for early vascular network formation, indicating that there may be differences in the role of macrophages between developmental and postdevelopmental angiogenesis [27]. Here, we sought to definitively assess the impact of early infiltrating macrophages on tissue proangiogenic VEGF-A expression, as well as the impact of macrophage VEGF-A expression on postdevelopmental arteriogenesis in the context of vascular injury and hind limb ischemia.

## 2. Methods

### 2.1. Resource Availability

**Lead Contact.** Further information and requests for resources and reagents should be directed to and will be fulfilled by the lead contact, Chris S. Mantsounga (chris_mantsounga@brown.edu).

**Materials Availability.** The *VEGF-A^fl/fl^* mice will be made available upon request to the corresponding author through use of a material transfer agreement (MTA) with Ocean State Research Institute, Inc. The MTA will be necessary to guide (1) general terms regarding how the mice will be used; (2) liabilities; (3) husbandry and shipping costs; (4) prevention of inappropriate distribution; (5) appropriate acknowledgement of the source; and (6) assurances that all planned animal subjects use is compliant with NIH and VA regulations.

**Data and code availability.** Microscopy data reported in this paper will be shared by the lead contact upon request. This paper does not report original code. Any additional information required to reanalyze the data reported in this paper is available from the lead contact upon request.

### 2.2. Experimental Model and Subject Details

**Animals.** All experiments were performed in accordance with the Providence VA Medical Center guidelines, and the Institutional Animal Care and Use Committee approved all housing protocols and experimental animal procedures. All animal procedures complied with the Office of Laboratory Animal Welfare and the National Institutes of Health Guide for Care and Use of Laboratory Animals. C57BL/6J (JAX, 000664) and FVB-Tg(Csf1r-cre/Esr1*)1Jwp/J (*Csf1r^mericremer^*; JAX, 019098) [28] mice were obtained commercially from The Jackson Laboratory. *VEGF-A^fl/fl^* mice were a generous gift from Frank Giordano. *VEGF-A^fl/fl^* mice were crossed with *Csf1r^meircremer^* mice to produce a myeloid-specific, tamoxifen-inducible *VEGF-A*-deletion in the context of an experimental model of acute hind limb ischemia. In general, animals 12–14 weeks of age were used in macrophage and hind limb ischemia studies. Male and female animals were used in equal numbers, and sex was analyzed as a biological variable by two-way ANOVA.

### 2.3. Method Details

**Induction of Cre Recombinase.** The mice were injected intraperitoneally with either vehicle control solution (100% ethanol in corn oil at a ratio of 20%:80%) or tamoxifen solution (1.0 mg per injection) for 10 days. The mice were then allowed to recover for one week before any further experiments were conducted.

**Clodronate liposome depletion of macrophages.** Liposomal control solution and liposomal solution containing clodronate (Fisher Scientific, Waltham, MA, USA, NC1361099) were injected intraperitoneally (250 µL/25 g/mouse) 3 days prior to flow cytometry or hindlimb ischemia. For acute hind limb ischemia studies, a second injection of 50 µL control or clodronate solution was administered intramuscularly in ischemic muscle tissue (gastrocnemius) immediately after surgery to limit any further residual macrophage recruitment to the ischemic site.

**Flow cytometry analysis.** Blood samples (70–100 μL), acquired by mandibular puncture of the mice, were collected before surgery (day −4) and days 3, 10, and 17 post-femoral-artery ligation. Absolute cell counts of CD45+ cells (2 μg/mL; BioLegend, San Diego, CA, USA, 103111) that co-stained for either CD3 (12.5 μg/mL; BioLegend, 100203), Ly6G (2 μg/mL; BioLegend, 127627), or CD115 (2 μg/mL; BioLegend, 135505) were stained and quantified using BD Trucount™ Tubes, according to the manufacturer’s instructions (BD Biosciences, Franklin Lakes, NJ, USA, 340334). Flow cytometry was carried out using a BD FACSAriaIIIu (BD Biosciences) cell sorter, according to Brown University Flow Cytometry and Cell Sorter Facility protocols, and data were analyzed with FlowJo™ v10.8 Software (BD Life Sciences, Franklin Lakes, NJ, USA).

**Hind limb ischemia model.** Femoral artery ligation surgeries were performed at the Providence VA Medical Center animal facility. Mice were anesthetized using inhaled isoflurane (1–3%) in an induction chamber and then transferred to the surgery platform, where they were kept under anesthesia through a fitted nose cone. The femoral artery was then ligated at two positions, spaced 5 mm apart, with one just below the inguinal ligament and the second distal to the superficial epigastric artery. All branches between the two ligatures were ligated, and the femoral artery segment was completely excised. Analgesic (Buprenorphine 0.05–0.1 mg/kg, Par Pharmaceuticals, Chestnut Ridge, NY, USA) was administered intraperitoneally at the time of surgery, four hours post-surgery, and then twice a day over the next three days. In some animals, early inflammatory assessment was performed on day 3 post-ligation, in which soleus and gastrocnemius ischemic muscle tissue was dissected, harvested, and segmented at the midpoint of the muscle for subsequent analysis. The muscle was then homogenized for subsequent TRIzol-based fractionation and isolation of DNA, RNA, and protein [29]. RNA was treated with deoxyribonuclease I (DNAse I; Millipore Sigma, Burlington, MA, USA, D7291) to remove genomic contaminant prior to reverse transcription. Immediately adjacent segments of the muscle tissue were fixed for cryomolding, followed by immunofluorescence histology. In some mice, mid-segments of the soleus and gastrocnemius tissue sections from both ischemic muscle and contralateral nonischemic control muscle were harvested on day 21 post-femoral-artery ligation for additional immunofluorescence histology.

**Laser Doppler blood flow imaging.** Mice were anesthetized by inhaled isoflurane (1–3%), as described above, and placed on a heating platform, where body temperature was kept at 37 ± 0.5 °C to minimize the influence of body temperature on blood flow imaging. Blood flow images of the hind paws were acquired using a Moor Laser Doppler Imager (MoorLDI2, Moor Instruments, Devon, UK). The data were analyzed with moorLDI image processing software (moorLDI V6.1) and reported as the ratio of blood flow in the ischemic to the non-ischemic, contralateral control hind paws. Blood flow was quantified before surgery (day −1), immediately after surgery (day 0), and at postoperative days 3, 7, 14, and 21.

**Histology and Immunofluorescence microscopy.** Muscle tissue (soleus and gastrocnemius), collected at either day 3 or 21 post-ligation, was placed in 30% sucrose for ≤1 week and embedded in optimal cutting temperature compound (Fisher Scientific, 23730571) in prelabeled cryomold squares (VWR, St. Louis, MO, USA, 25608916), followed by dry ice for 10 to 15 min. Tissue was sectioned at an 8 µm thickness by cryostat (Leica Biosystems, Nussloch, Germany, CM3050 S), and then slides with sections were dried at room temperature for 1 to 2 h before permeabilization, blocking, and immunostaining. Sections were incubated with CD31-FITC (5 μg/mL; Fisher Scientific, 11-0311-85), CD68-APC (2 μg/mL; BioLegend, 137008), α-SMA-Cy3 (10–15 μg/mL; Millipore Sigma, C6198), and 4′,6-Diamidino-2-phenylindole dihydrochloride (DAPI) (Thermo Fisher, Waltham, MA, USA, P36931). Using a Nikon Eclipse 80i inverted microscope with Nikon Plan Apochromat 10× and 20× objectives lens (numerical aperture 0.3), three to four fluorescence images were acquired per animal and then averaged in regions of cellular infiltration denoted by DAPI staining. Quantification of cell types was expressed as a ratio of each DAPI+ cell co-staining for CD68, CD31, or SMA relative to the total DAPI-stained cells in the field. All subsequent analyses were performed using Image J software (Version 2.0.0-rc-69/1.52p) [30].

**Bone marrow-derived macrophage (BMDM) differentiation.** BMDMs were differentiated in culture from femur and tibia primary bone marrow cells using validated protocols [14,31]. Bones were dissected, cleaned, and disinfected in 70% ethanol. Bone marrow was flushed from the bone and washed with fully supplemented RPMI 1640 medium (10% fetal bovine serum, 10 mmol/L HEPES pH 7.4, 2 mmol/L L-glutamine, 100 units/mL penicillin, 10 μg/mL streptomycin, and 50 μM 2-ME [2-mercaptoethanol]; Fisher Scientific, 11875085). Red blood cells were removed by ammonium-chloride potassium lysis buffer, followed by centrifugation. The remaining cells were sequentially filtered at 70 μm and then 40 μm, and then cells were plated at a density of 3.5 × 10^6^ cells/10 cm Petri dish in fully supplemented RPMI with 30% (*vol*/*vol*) L929 cell-conditioned medium. Differentiation to macrophages occurred over 6–7 days and was confirmed by >98% expression of F4/80 or CD68. Non-adherent cells were washed away with PBS, while adherent cells were recovered by gentle pipetting in PBS with 1 mM EDTA. The phenotypic polarization of BMDMs was driven by incubation with the combination of LPS (100 ng/mL; Sigma-Aldrich, L2630) and IFN-γ (50 ng/mL; BioLegend, 575304) or with the combination of IL-4 (10 ng/mL; BioLegend, 574304) and IL-13 (10 ng/mL; BioLegend, 575904) for 18 h. For the BMDM transplantation experiments, BMDMs (5 × 10^5^ cells) from WT control or mVEGF-A KO were injected intravenously into clodronate liposome macrophage-depleted mVEGF-A KO animals eight hours following femoral artery ligation.

**RT-qPCR Analysis.** Total RNA from ischemic muscle tissue (100–150 mg) was extracted using Trizol (Fisher Scientific, 15596026). Total RNA from BMDMs (1.5 million cells/mL) was extracted from RNA lysis buffer (ZYMO Research, Irvine, CA, USA, R1058), according to the manufacturer’s protocol. RNA concentration was measured using Nanodrop One (Thermo Fisher). First-strand complementary DNA was synthesized with the iScript cDNA synthesis Kit (Bio-Rad, Hercules, CA, USA, 1708891). Equal amounts of RNA (150 ng) were used as templates in each reaction. Quantitative PCR was performed with SSoAdvanved UnivSYBR Supermix (Bio-Rad, 1725274) on the StepOne Plus PCR machine (Applied Biosystems, Waltham, MA, USA) using the following primers: HPRT sense: 5′-GACCGGTCCCGTCATGCCGA-3′ antisense: 5′-TGGCCTCCCATCTCCTCCATGACA-3′; GAPDH sense: 5′-GTGTGAACGGATTTGGCCG-3′ antisense: 5′-GTGATGGGCTTCCCGTTGAT-3′; IL-1β sense: 5′-AAAGATGAAGGGCTGCTTCC-3′ antisense: 5′-GTCCACGGGAAAGACACAGG-3′; VEGF-A sense: 5′-ACTGGACCCTGGCTTTACTGC-3′ antisense: 5′-TGATCCGCATGATCTGCATGGTG-3′; VEGF-A_165_a sense: 5′-CAGAAAATCACTGTGAGCCTTGTT-3′ antisense: 5′-CTTGGCTTGTCACATCTGCAA-3′; VEGF-A_165_b sense: 5′-CAGAAAATCACTGTGAGCCTTGTT-3′ antisense: 5′-CTTTCCGGTGAGAGGTCTGC-3′; Ang2 sense: 5′-TCATCACCCAACTCCAAGAGC-3′ antisense: 5′-ACGTCCATGTCACAGTAGGC-3′; from Integrated DNA Technologies (IDT). All measurements were carried out in triplicate with HPRT and GAPDH as housekeeping genes in the BMDMs and muscle tissue, respectively.

**Immunoblotting.** Protein from ischemic muscle tissue (100–150 mg) was extracted using Trizol (Fisher Scientific, 15596026). Protein was collected in Laemmli sample buffer (Bio-Rad, 1610737) containing 2-βME, then boiled at 95 °C and aliquoted. The aliquots from tissue were separated by Tris/Glycine/SDS–PAGE (Bio-Rad, 1610732) and blotted onto PVDF membranes (Bio-Rad, 1704157). The membranes were blocked in a fluorescent blocking buffer (Rockland Immunochemicals, Limerick, PA, USA, MB070) and probed at 4 °C overnight with antibodies against VEGF-A (1 μg/mL; Abcam, Cambridge, UK, ab46154), cleaved mature IL-1β (1 μg/mL; Cell Signaling Technologies, Danvers, MA, USA, 52718), and β-Actin (0.2 μg/mL; Santa Cruz Biotechnology, Dallas, TX, USA, sc-47778). After washing, the membranes were incubated with fluorescent secondary antibodies (0.4 μg/mL; Thermo Fisher, A21088; and 2 μg/mL; LI-COR, Lincoln, NE, USA, 926-32212). The proteins were quantified by densitometry, using the LI-COR Imager (LI-COR, Odyssey CLx) and normalization to β-Actin.

**Enzyme-linked immunosorbent assay (ELISA).** Quantitation of IL-1β (BioLegend, 432604) and VEGF-A (R&D Systems, Minneapolis, MN, USA, DY493) concentrations in BMDM culture media supernatant was carried out by ELISA, according to the manufacturer protocols. Each measure represents the average of experimental triplicates from a single animal.

**Statistical Analysis**. All statistical data were analyzed with the use of Prism 10 (GraphPad, Boston, MA, USA, v10.1.0). The results are presented as the mean (SD) for continuous variables with normal distribution or equal variance and as the median (interquartile range) for continuous variables without normal distribution. The *t*-test was used to compare normally distributed continuous variables between two independent groups. Differences between multiple groups were assessed by ANOVA, followed by Tukey’s post hoc multiple comparisons test. The Mann–Whitney *U* test was used for continuous variables not normally distributed. Male and female animals were used in equal numbers, and sex was analyzed as a biological variable by two-way ANOVA.

## 3. Results

### 3.1. Macrophages Are Required for Inflammatory Arteriogenesis in Response to Acute Hind Limb Ischemia

**Figure 1:**
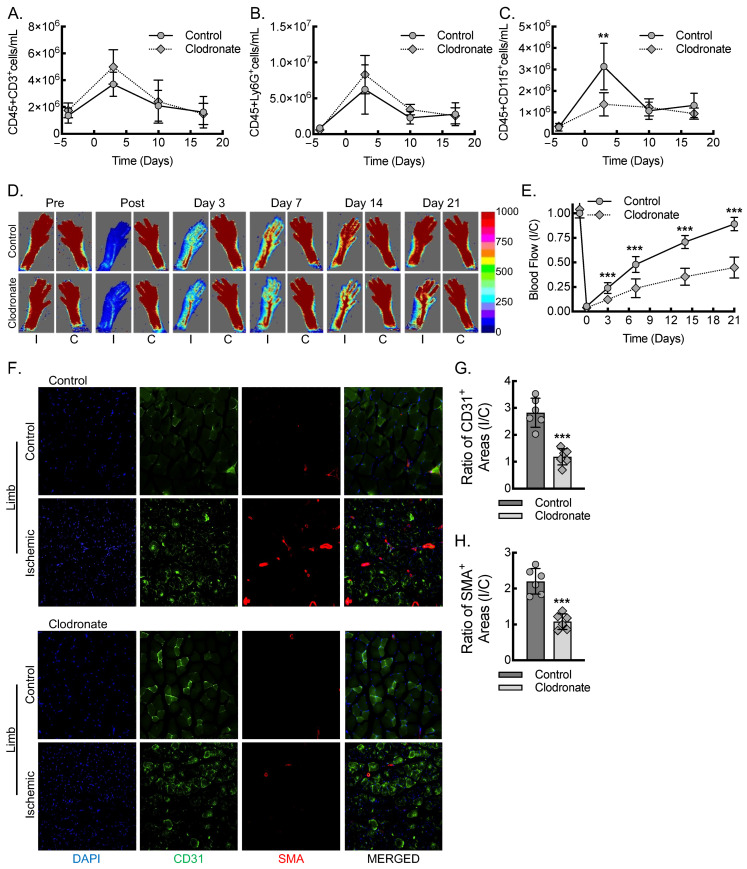
Macrophages are required for inflammatory arteriogenesis in response to acute hind limb ischemia. (**A**–**C**) Flow cytometry assessment of CD45^+^CD3^+^ (**A**), CD45^+^Ly6G^+^ (**B**), and CD45^+^CD115^+^ (**C**) cells, before and after femoral artery ligation, at day 0 (**, *p* < 0.005; relative to control by multiple *t*-tests; *n* = 12, 6 males and 6 females). (**D**) Laser Doppler images of flow in the ischemic (I) and contralateral control (**C**) hind limbs of control or clodronate liposome-treated mice at indicated time points, before and after femoral artery ligation, along with quantitative analysis (**E**) (***, *p* < 0.0001; compared between control and clodronate at each timepoint by ANOVA; *n* = 12, 6 males and 6 females). (**F**) Immunofluorescence micrographs of ischemic and contralateral control muscle tissue at day 21 post-femoral-artery ligation from either control or clodronate-treated mice, along with quantitation of the ratio of CD31^+^ areas (**G**) or SMA^+^ areas (**H**) between ischemic and contralateral control limbs (***, *p* < 0.0001 by *t*-test; *n* = 6 mice total, 3 males and 3 females). Bar, 100 microns. Data, mean ± SD.

To quantify the impact of monocyte/macrophage populations on proangiogenic VEGF-A expression and inflammatory arteriogenesis in the setting of acute hind limb ischemia, we first carried out a clodronate liposome macrophage depletion, followed by femoral artery ligation in C57BL/6J mice [13,14,32,33,34]. We validated the efficacy and specificity of clodronate liposomes for the depletion of monocytes/macrophages relative to the control liposomes using flow cytometry on whole blood stained for circulating leukocyte markers, collected at multiple time points prior to and then 3, 10, and 17 days post-femoral-artery ligation (Figure 1A–C; Supplemental Figure S1). The absolute quantitation of T cells (CD45^+^CD3^+^), neutrophils (CD45^+^Ly6G^+^), and monocytes/macrophages (CD45^+^CD115^+^) was carried out, and each population demonstrated a peak in circulation at day 3 post-ligation. There were no significant changes in the day 3 peaks of CD45^+^CD3^+^ cells or CD45^+^Ly6G^+^ cells with clodronate liposome treatment relative to those of the control liposome treatment. However, flow cytometry for circulating monocyte/macrophage populations, marked as CD45^+^CD115^+^ cells, demonstrated a 60% reduction in the clodronate liposome group relative to that of the control liposome group at day 3 post-ligation.

We sought to understand whether this loss of monocyte/macrophage populations would significantly impair perfusion recovery in a femoral artery ligation model of acute hind limb ischemia. Clodronate liposome depletion of macrophages resulted in a 50% decreased blood flow recovery, indicative of impaired angiogenesis and arteriogenesis (Figure 1D,E). Comparable blood flow recovery between male and female mice was noted in both the control and clodronate liposome treatment groups when analyzing sex as a biological variable using ANOVA (Supplemental Figure S2). To assess the proliferation of blood vessels consequent to angiogenesis and arteriogenesis, we quantified the area of CD31^+^ vessels and smooth muscle actin positive (SMA^+^) vessels in the soleus and gastrocnemius muscles 21 days after femoral artery ligation. We then calculated the ratio of ischemic to non-ischemic contralateral control muscle vessel area in the control and clodronate liposome-treated mice (Figure 1F–H). We identified nearly a three-fold increase in CD31^+^ vessel area in the ischemic limb compared to the contralateral control limb in the control mice relative to the clodronate liposome-treated mice. Additionally, we found an approximately two-fold increase in the ratio of SMA^+^ vessel area, a marker of new arterial growth, in the ischemic compared to the contralateral control limb in the control mice relative to that of the clodronate liposome-treated mice (Figure 1F–H). In summary, the data indicate that clodronate liposome treatment of mice transiently depletes macrophages, leading to consequent impaired angiogenesis and arteriogenesis responses to acute hind limb ischemia.

### 3.2. Macrophages Are Required for Endothelial Cell Recruitment and Adequate Tissue VEGF-A Levels During Early Inflammatory Angiogenesis

**Figure 2:**
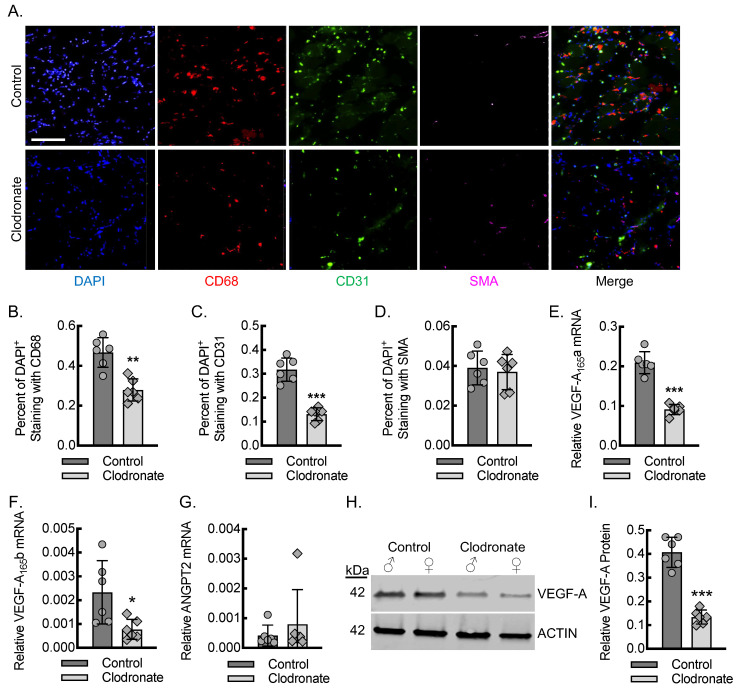
Macrophages are required for endothelial cell recruitment and tissue VEGF-A levels during early inflammatory angiogenesis. (**A**) Immunofluorescence micrographs of ischemic muscle tissue at day 3 post-femoral-artery ligation from either control or clodronate-treated mice, along with quantitation of DAPI^+^CD68^+^ (**B**), DAPI^+^CD31^+^ (**C**), and DAPI^+^SMA^+^ (**D**) cells (**, *p* < 0.001; ***, *p* < 0.0001 by *t*-test; *n* = 6 mice, 3 males and 3 females). Bar, 100 microns. (**E**–**G**) RT-qPCR for relative VEGF-A_165_a (**E**), VEGF-A_165_b (**F**), and ANGPT2 (**G**) mRNA expression from ischemic muscle of animals treated as in (**A**) (*, *p* < 0.05; ***, *p* < 0.0001 by *t*-test; *n* = 6 mice total, 3 males and 3 females). (**H**) Representative VEGF-A immunoblots from ischemic muscle tissue of animals treated as in (**A**), with each lane containing muscle lysate from a separate animal, along with quantification (**I**) of β-actin-normalized protein (***, *p* < 0.0001 by *t*-test; *n* = 6 mice, 3 males and 3 females). Data, mean ± SD.

To quantitate the impact of macrophages on endothelial cell recruitment for angiogenesis during the early stages of inflammation after acute hind limb ischemia, we carried out immunofluorescence staining of the ischemic soleus and gastrocnemius muscle tissue on day 3 post-femoral-artery ligation from mice treated with clodronate and control liposomes (Figure 2A–D). The percent of 4′,6-diamidino-2-phenylindole (DAPI) positive cells co-staining for the macrophage marker CD68 was reduced in the mice undergoing clodronate liposome treatment by about 50% relative to that of the mice receiving control liposome treatment, consistent with the flow cytometry data for blood. The percentage of DAPI^+^ cells that co-stained positive for the endothelial maker CD31 was also significantly reduced by about 50% in the mice undergoing clodronate liposome treatment relative to that of the control liposome treatment. The percentage of DAPI^+^ cells that co-stained positive for SMA was comparable between the mice treated with clodronate and the control liposomes, consistent with the similar baseline arteriole density between the mice.

To confirm that macrophages have an impact on the total levels of VEGF-A in ischemic muscle tissue, we quantitated VEGF-A and ANGPT2 mRNA and VEGF-A protein using primer-specific RT-qPCR and immunoblotting, respectively, from ischemic soleus and gastrocnemius muscle tissue lysates (Figure 2E–I). On day 3 post-femoral-artery ligation, there was a reduction in proangiogenic VEGF-A (VEGF-A_165_a) isoform mRNA of about 60% in the ischemic limbs from the clodronate liposome-treated relative to that of the control liposome-treated mice. The quantitation of mRNA specific for the antiangiogenic VEGF-A isoform VEGF-A_165_b was also reduced by about 60%, indicating that both isoforms were impacted by the clodronate liposome depletion model. The quantitation of ANGPT2 mRNA revealed a comparable expression between clodronate liposome-treated relative to that of the control liposome-treated mice. Protein immunoblotting of the ischemic soleus and gastrocnemius muscle tissue lysates revealed about a 66% reduction in VEGF-A protein levels in the clodronate liposome-treated relative to that of the control liposome-treated mice, demonstrating consistency between the measured mRNA and protein levels.

### 3.3. Myeloid VEGF-A-Deletion Leads to Reduced Macrophage VEGF-A Expression and Impaired Blood Flow Recovery Secondary to Decreased Inflammatory Angiogenesis and Arteriogenesis

**Figure 3:**
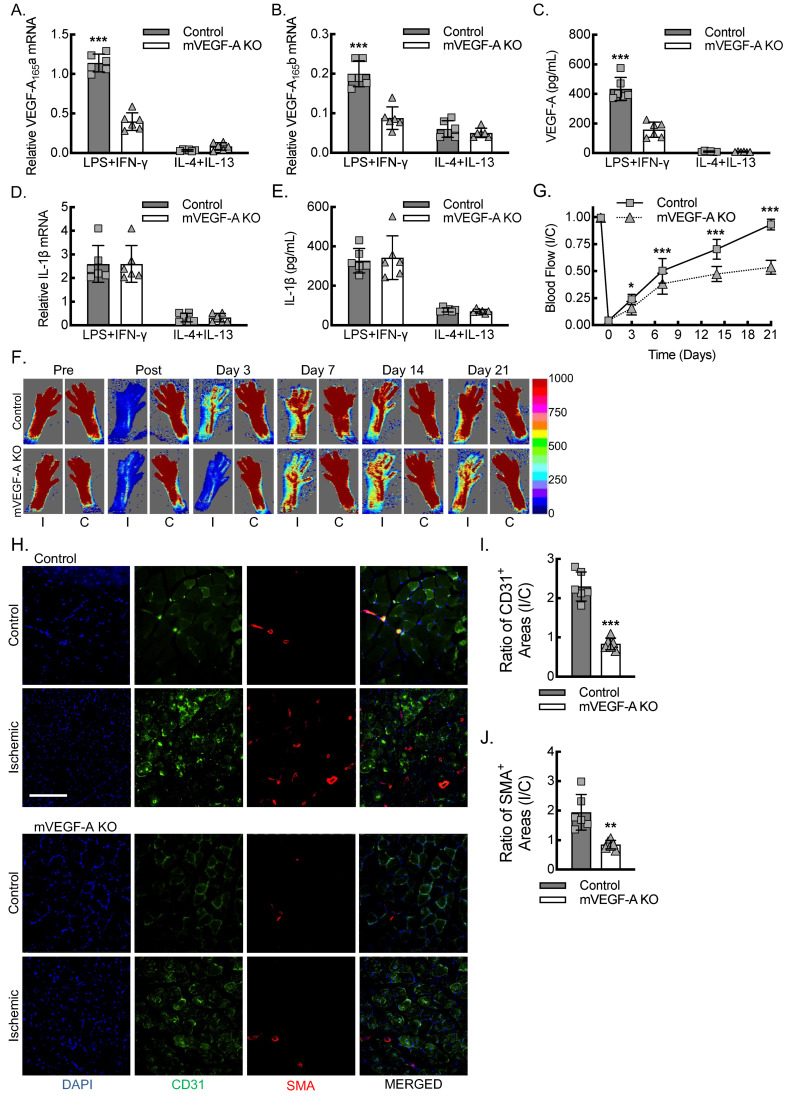
Myeloid *VEGF-A*-deletion leads to reduced macrophage VEGF-A expression and impaired blood flow recovery secondary to decreased inflammatory angiogenesis and arteriogenesis. (**A**) Primary BMDMs from control or mVEGF-A KO mice were treated with either LPS + IFN-γ or IL-4 + IL-13 for 24 h, followed by RT-qPCR for relative VEGF-A_165_a mRNA expression (***, *p* < 0.0001 compared to all others by ANOVA; *n* = 6 mice total, 3 males and 3 females). (**B**) BMDMs treated as in (**A**), followed by RT-qPCR for relative VEGF-A_165_b mRNA expression (***, *p* < 0.0001 compared to all others by ANOVA; *n* = 6 mice total, 3 males and 3 females). (**C**) BMDMs treated as in (**A**), followed by ELISA on culture media for secreted VEGF-A protein (***, *p* < 0.0001 compared to all others by ANOVA; *n* = 6 mice total, 3 males and 3 females). (**D**) Primary BMDMs treated as in (**A**), followed by RT-qPCR for relative IL-1β mRNA expression (*n* = 6 mice total, 3 males and 3 females). (**E**) BMDMs treated as in (**A**), followed by ELISA on culture media for secreted mature IL-1β protein (*n* = 6 mice total, 3 males and 3 females). (**F**) Laser Doppler images of blood flow in the ischemic (**I**) and contralateral control (**C**) hind limbs of control or mVEGF-A KO mice at indicated time points, before and after femoral artery ligation, along with quantitative analysis (**G**) (*, *p* < 0.05; ***, *p* < 0.0001; compared between control and mVEGF-A KO at each timepoint by ANOVA; *n* = 12, 6 males and 6 females). (**H**) Immunofluorescence micrographs of ischemic and contralateral control muscle tissue at day 21 post-femoral-artery ligation from either control or mVEGF-A KO mice, along with quantitation of the ratio of CD31^+^ areas (**I**) or SMA^+^ areas (**J**) between ischemic and contralateral control limbs (**, *p* = 0.0016; ***, *p* < 0.0001 by *t*-test; *n* = 6 mice total, 3 males and 3 females). Bar, 100 microns. Data, mean ± SD.

We sought to quantitate the impact of macrophage VEGF-A expression on perfusion recovery in the femoral artery ligation model of hind limb ischemia. To develop an animal model of conditional myeloid *VEGF-A*-deletion, we crossed a mouse strain containing LoxP flanked exon 3 of *VEGF-A* (*VEGF-A^fl/fl^*) with the FVB-Tg(Csf1r-cre/Esr1*)1Jwp/J strain of mice that express the tamoxifen-inducible MeriCreMer fusion protein under control of the promoter, colony stimulating factor 1 receptor (*Csf1r^mericremer^*; JAX, 019098) [28,35]. Next, we validated the impact of myeloid *VEGF-A*-deletion (mVEGF-A KO) on VEGF-A and IL-1β expression in bone marrow-derived macrophages (BMDMs) under conditions of LPS + IFN-γ (inflammatory) or IL-4 + IL-13 (alternatively activated) polarization (Figure 3A–E). By primer-specific RT-qPCR, inflammatory macrophages expressed the highest levels of mRNA for both VEGF-A_165_a and VEGF-A_165_b in the LPS + IFN-γ treated BMDMs, and mVEGF-A KO led to about a 66% reduction in both isoforms. Quantitation of VEGF-A protein secreted into the culture media by ELISA revealed a comparable pattern, whereby there was about a 40-fold increase in VEGF-A protein secretion from BMDMs polarized with LPS + IFN-γ relative to IL-4 + IL-13, and mVEGF-A KO led to about a 66% reduction in the secretion of VEGF-A protein. Of note, IL-1β expression was the highest under conditions of stimulation with LPS + IFN-γ, and BMDMs from mVEGF-A KO exhibited no change in the expression of IL-1β mRNA or protein relative to BMDMs from the control animals.

Relative to the control mice, the mVEGF-A KO mice demonstrated decreased blood flow over the three weeks following femoral artery ligation, consistent with impaired angiogenesis and arteriogenesis (Figure 3G,F). Sex was analyzed as a biological variable using ANOVA, and we found no evidence of sex-specific changes in blood flow recovery between male and female mice in either the control or mVEGF-A KO mice (Supplemental Figure S3). To assess the proliferation of blood vessels consequent to angiogenesis and arteriogenesis, we quantified the area of CD31^+^ vessels and smooth muscle actin positive (SMA^+^) vessels in the soleus and gastrocnemius muscles 21 days after femoral artery ligation. We then calculated the ratio of ischemic to non-ischemic contralateral control muscle vessel area in the control and mVEGF-A KO mice (Figure 3H–J). There was an almost three-fold increase in the ratio of CD31^+^ vessel area in the ischemic to control limb in the control mice but no significant increase in the mVEGF-A KO mice. The ratio of SMA^+^ vessel area, as a marker of new arterial growth, in ischemic limb to contralateral control limb revealed about a two-fold increase in the control mice but essentially no change in the mVEGF-A KO mice. In summary, the data indicate that myeloid *VEGF-A*-deletion leads to reduced VEGF-A expression from the inflammatory macrophages and impaired blood flow recovery in the experimental PAD model consequent to failed angiogenesis and arteriogenesis responses to acute hind limb ischemia.

### 3.4. Myeloid VEGF-A Expression Is Required for Endothelial Cell Recruitment and Sufficient Tissue VEGF-A Levels During Early Inflammatory Angiogenesis

**Figure 4:**
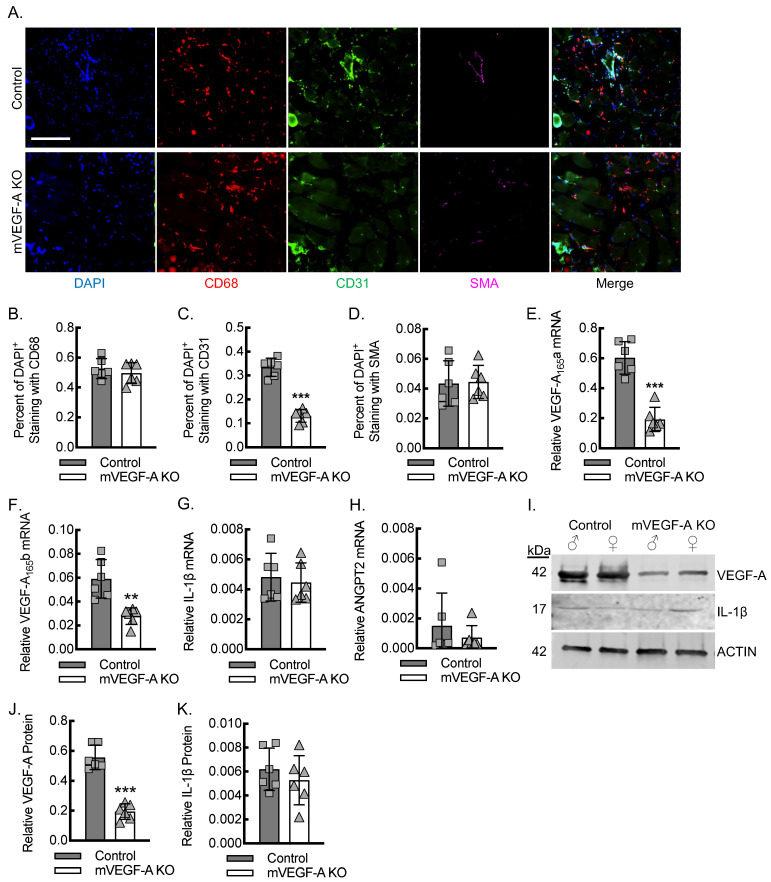
Myeloid VEGF-A expression is required for endothelial cell recruitment and sufficient tissue VEGF-A levels during early inflammatory angiogenesis. (**A**) Immunofluorescence micrographs of ischemic muscle tissue at day 3 post-femoral-artery ligation from either control or mVEGF-A KO mice, along with quantitation of DAPI^+^CD68^+^ (**B**), DAPI^+^CD31^+^ (**C**), and DAPI^+^SMA^+^ (**D**) cells (***, *p* < 0.0001 by *t*-test; *n* = 6 mice, 3 males and 3 females). Bar, 100 microns. (E-H) RT-qPCR for relative VEGF-A_165_a (**E**), VEGF-A_165_b (**F**), IL-1β (**G**), and ANGPT2 (**H**) mRNA expression from ischemic muscle of animals treated as in (**A**) (**, *p* = 0.0015; ***, *p* < 0.0001 by *t*-test; *n* = 6 mice total, 3 males and 3 females). (**I**) Representative VEGF-A and IL-1β immunoblots from ischemic muscle tissue of animals treated as in (**A**), with each lane containing muscle lysate from a separate animal, along with quantification (**J**,**K**) of β-actin-normalized protein (***, *p* < 0.0001 by *t*-test; *n* = 6 mice, 3 males and 3 females). Data, mean ± SD.

To quantitate the impact of myeloid VEGF-A expression on endothelial cell recruitment for angiogenesis during the early stages of inflammation after acute hind limb ischemia, we carried out the immunofluorescence staining of ischemic soleus and gastrocnemius muscle tissue on day 3 post-femoral-artery ligation from control and mVEGF-A KO mice (Figure 4A–D). The percentage of DAPI positive cells co-staining for the macrophage marker CD68 was comparable between the control and mVEGF-A KO mice, indicating that myeloid *VEGF-A*-deletion had no impact on macrophage recruitment to the site of inflammation. However, the percentage of DAPI^+^ cells that co-stained positive for the endothelial maker CD31 was significantly reduced by about 65% in the mVEGF-A KO relative to that of the control mice. The percent of DAPI^+^ cells that co-stained positive for SMA was comparable between the control and VEGF-A KO mice.

To confirm that myeloid VEGF-A expression impacted the total levels of VEGF-A in ischemic muscle tissue, we quantitated VEGF-A and ANGPT2 mRNA using primer-specific RT-qPCR and VEGF-A protein by immunoblotting, using ischemic soleus and gastrocnemius muscle tissue lysates (Figure 4E–J). On day 3 post-femoral-artery ligation, there was a reduction in proangiogenic VEGF-A_165_a isoform mRNA of about 66% in the ischemic limbs from mVEGF-A KO mice relative to that of the control mice. Quantitation of VEGF-A_165_b mRNA also revealed about a 50–60% reduction in mVEGF-A KO mice relative to that of the control mice. The quantitation of relative IL-1β mRNA expression revealed comparable expression between the control and mVEGF-A mice, indicating that the reductions in VEGF-A expression were independent of IL-1β. The quantitation of ANGPT2 mRNA revealed comparable expression between the control and mVEGF-A KO mice. Protein immunoblotting of the ischemic soleus and gastrocnemius muscle tissue lysates revealed about a 66% reduction in VEGF-A protein levels in mVEGF-A KO mice relative to that of the control mice, demonstrating consistency between the measured mRNA and protein levels. Of note, immunoblotting of ischemic muscle tissue lysates for mature IL-1β protein levels revealed comparable expression between the control and mVEGF-A KO mice, supporting the phenotypic changes demonstrated by myeloid *VEGF-A*-deletion to be independent of IL-1β expression.

### 3.5. Adoptive Transfer of Macrophages Polarized Toward an Inflammatory State Is Sufficient to Rescue Blood Flow Recovery in Myeloid VEGF-A-Deleted Mice Undergoing Hind Limb Ischemia

**Figure 5:**
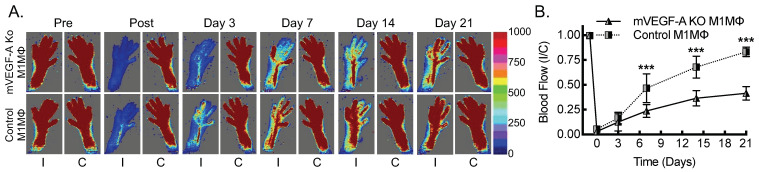
Adoptive transfer of macrophages polarized toward an inflammatory state is sufficient to rescue blood flow recovery in myeloid *VEGF-A*-deleted mice undergoing hind limb ischemia. (**A**) Laser Doppler images of flow in the ischemic (I) and contralateral control (C) hind limbs of mVEGF-A KO mice that underwent clodronate liposome macrophage depletion, followed by transplant of LPS + IFN-γ-treated BMDMs from either wild-type (Control Mϕ) or *VEGF-A*-deleted (mVEGF-A KO Mϕ) mice, along with quantitative analysis (**B**) (***, *p* ≤ 0.0001 compared between control Mϕ and mVEGF-A Mϕ for each timepoint by ANOVA; *n* = 8 mice total, 4 males and 4 females).

The specificity of the *Csf1r* promoter-driven Cre recombinase system may be confounded by its expression in neutrophils, NK cells, and certain subsets of T cells [36]. To address this concern in the mVEGF-A KO system, we performed clodronate liposome macrophage depletion, followed by intravenous injection of cultured BMDMs that were polarized toward an inflammatory state with LPS + IFN-γ to confirm that macrophages are sufficient to rescue the myeloid *VEGF-A*-deletion phenotype in the experimental PAD model. Briefly, mVEGF-A KO mice underwent femoral artery ligation in the setting of clodronate liposome macrophage depletion, followed by a transplant of either autologous mVEGF-A KO BMDMs or wild-type control BMDMs, and they were followed over 3 weeks for blood flow recovery using laser Doppler (Figure 5). The mVEGF-A KO mice that received autologous BMDMs demonstrated an approximately 40% perfusion recovery, consistent with the established myeloid *VEGF-A*-deletion phenotype. The mVEGF-A KO mice that received wild-type control BMDMs demonstrated blood flow recovery that was comparable to that of the wild-type control mice, with approximately 85% recovery within 21 days. When analyzing sex as a biological variable using ANOVA, we found no evidence of sex-specific differences in blood flow recovery between male and female mice in this BMDM transplant rescue study (Supplemental Figure S4). In summary, inflammatory BMDMs were sufficient to rescue the phenotype imparted by myeloid *VEGF-A*-deletion.

## 4. Discussion

There is growing appreciation for inflammatory signaling as an important mechanism that contributes to postdevelopmental angiogenesis and arteriogenesis responses to injury complementary to other well-established mechanisms like hypoxia, shear stress signaling, and the mobilization of bone marrow-derived progenitor cell populations [14,26,27,28,32,37,38,39,40,41]. Multiple cell types contribute to postdevelopmental angiogenesis, but inflammatory cells secrete a number of orchestrating chemokines, cytokines, and growth factors that may display context-dependent proangiogenic or antiangiogenic effects [11,14,26,42,43]. Here, we demonstrated that early infiltrating macrophages recruited to the ischemic muscle tissue within the first three days following vascular injury by femoral artery ligation are required to orchestrate capillary and arterial vessel growth that sets the stage for blood flow recovery to the limb weeks later. We further established that early infiltrating macrophages represent a major source of VEGF-A expression, including the proangiogenic isoform, which is required for endothelial cell recruitment and successful inflammatory-mediated angiogenesis and arteriogenesis.

In the setting of vascular injury, the chemokine CCL2 (MCP-1) plays a major role in recruiting inflammatory monocytes to the infarcted tissue via its receptor CCR2 [15,16]. Previously, we found that CCR2 expression is highly upregulated on the surface of inflammatory monocytes/macrophage subsets, characterized by high levels of CD14 expression and low levels of the cluster of differentiation molecule CD16 (FcγRIII) [13]. Inflammatory monocytes/macrophages appear to be a major source of proangiogenic VEGF-A tissue levels, in part via the CCL2-CCR2 signaling-mediated posttranscriptional regulation of VEGF-A mRNA but also through IL-1β-IL-1R signaling-mediated transcription of VEGF-A [13,14]. This challenges certain paradigms that inflammatory macrophages primarily delay or suppress angiogenesis-dependent healing, while only alternatively activated macrophages promote wound healing and vascular repair [19,20,44,45,46,47]. These findings may reflect context-dependent differences in macrophage responses with respect to different experimental models but may also reflect the dynamic plasticity of macrophage subsets and the challenge of kinetically parsing out individual subset responses in tissue [48].

Here, we confirmed the importance of early infiltrating macrophages as a source of ischemic tissue VEGF-A expression for recruitment of endothelial cells and consequent angiogenesis and arteriogenesis during the femoral artery ligation model of acute hind limb ischemia. Clodronate liposome depletion of macrophage populations was confirmed in the blood and in the ischemic muscle tissue using macrophage markers CD115 and CD68, respectively. Clodronate liposome depletion of macrophage populations led to decreased VEGF-A expression in ischemic tissue, decreased endothelial cell (CD31^+^) recruitment, and impaired blood flow recovery, with reduced angiogenesis and arteriogenesis. It is important to acknowledge the limitations of CD115 and CD68 as purely macrophage-specific markers. CD115 (*Csf1r*) may be expressed on some neutrophils and some subsets of T cells, potentially confounding in vivo findings [36]. Moreover, the specificity of CD68 for macrophages has been called into question, as mesenchymal-derived cells may also upregulate CD68 expression in response to inflammation [49,50]. While clodronate liposome treatment is established to deplete macrophage populations, clodronate liposomes also stun neutrophils and deplete other myeloid cells, including monocytes and dendritic cells, and thus, phenotypes may be reflective of broad myeloid effects, including impaired neutrophil function [51]. Our prior research in this area has demonstrated that over 98% of CD68 positive cells found in the ischemic muscle tissue are macrophages, as confirmed by multiple overlapping cellular markers, including CD115, F4/80, and macrophage transplant studies [14]. The adoptive transfer of macrophages presented in this current study also allowed for confirmation that macrophages were sufficient to rescue the blood flow recovery conferred by clodronate liposome treatment. However, this does not rule out the possibility of other systemic effects from clodronate liposome treatment. In fact, we also found a modest trend toward increased populations of neutrophils and T-cells with clodronate liposomes, potentially consequent to the decreased macrophage population. These increases were not significant and had no impact on blood flow recovery.

Prior work in the area of developmental vascularization demonstrated that macrophage VEGF-A expression was not required for early vascular network formation [27]. Here, we confirm that macrophage-derived VEGF-A expression is required for endothelial cell recruitment to the ischemic limb and blood flow recovery, with histological evidence of both angiogenesis and arteriogenesis. While we do not negate the importance of other cellular sources of VEGF-A, including endothelial cells fibroblasts and vascular smooth muscle cells, we have identified macrophages to be responsible for about two-thirds of the VEGF-A expression in ischemic muscle tissue on day 3 post-femoral-artery ligation. This may reflect differences between developmental and postdevelopmental angiogenesis in the context of inflammation. We previously demonstrated that pro-angiogenic VEGF-A expression, endothelial cell recruitment to the site of inflammation, and consequent postdevelopmental arteriogenesis in the PAD model are all, in fact, completely dependent upon the macrophage expression of IL-1β [14]. However, we had not previously confirmed that IL-1β acts through VEGF-A to recruit endothelial cells required for revascularization. Prior studies using a model involving the injection of recombinant IL-1β, with or without an inhibitory antibody, to VEGF-A demonstrated that recombinant IL-1β increased the recruitment of endothelial cell precursors to the ischemic limb in a VEGF-A-dependent manner, but the study design did not allow for identification of the primary cellular source(s) of IL-1β or VEGF-A [26]. Here, we demonstrated that macrophage IL-1β expression is not impacted by *VEGF-A*-deletion. In fact, we confirmed through macrophage-specific *VEGF-A*-deletion and BMDM transplant rescue that in the context of physiologic IL-1β levels, macrophage VEGF-A expression is required for endothelial cell recruitment and blood flow recovery consequent to angiogenesis and arteriogenesis in the experimental PAD model.

ANGPT2 is primarily produced by endothelial cells and hematopoietic-derived cells and is expressed at sites of vascular remodeling [52,53]. ANGPT2 has been shown to both promote and destabilize vessels in the remodeling process, and the way in which this balance is coordinated remains unclear. In part, ANGPT2 expression appears to be involved in vascular pruning and maturation, as well as in the resolution of inflammation by reducing VEGF-A expression and reducing MCP-1-induced macrophage migration to the site of ischemia [54]. We assessed the expression of ANGPT2 mRNA in the ischemic muscle tissue 3 days post-femoral-artery ligation. We found that the expression was relatively low when compared with that of proangiogenic VEGF-A_165_a, and that the expression was not impacted by macrophage depletion or macrophage *VEGF-A*-deletion. This may indicate that the kinetics of ANGPT2 expression are quite different from those of VEGF-A, or that macrophages are not a major source of ANGPT2 during the early stages of inflammation and angiogenesis following vascular injury. Future studies assessing the crosstalk between VEGF-A and ANGPT2 in postdevelopmental angiogenesis and arteriogenesis may help to further elucidate the balance between these two signaling mechanisms in the regulation of vascular remodeling.

We have demonstrated that the loss of VEGF-A from early infiltrating macrophages led to significant impairments in angiogenesis and arteriogenesis in the experimental PAD model. We do not rule out the importance of other growth or hypoxia factors in the promotion of inflammatory angiogenesis and arteriogenesis in response to acute vascular injury. In particular, we have previously assessed hypoxia-induced factor 1-alpha (HIF-1α), a critical hypoxia sensor, and while it was activated in response to hind limb ischemia, HIF-1α was insufficient to compensate for the role of IL-1β in promoting macrophage VEGF-A expression [14]. Alternatively, while the loss of fibroblast growth factor 2 (FGF2) also resulted in impaired functional recovery of ischemic limbs, *FGF2^−/−^* mice demonstrated comparable increases in capillary and arteriolar density relative to those of the control mice in the femoral artery ligation model, suggesting that the mechanism of action of FGF2 is likely centered on skeletal muscle recovery [55]. While signaling via HIF-1α and FGF2 are clearly critical complementary mechanisms supporting recovery from acute hind limb ischemia, they do not appear to be sufficient to account for the loss of inflammatory-driven VEGF-A expression in macrophages.

While we demonstrated the ability of phenotypically inflammatory macrophages to rescue blood flow recovery in mVEGF-A KO mice, we did not analyze the survivability of these macrophages over time, their long-term impact on the resident macrophage population, or their phenotypic plasticity throughout the healing process. Future kinetic studies will be required to assess the long-term survival of transplanted macrophages, along with any dynamic plasticity to their phenotypic profiles in order to assess the practical applicability of this treatment to cell-based therapeutic angiogenesis strategies.

A limitation of our work and that of the field in general is the inability to discriminate the respective contributions of the VEGF-A isoforms, VEGF-A_165_a and VEGF-A_165_b. Clinical PAD has been associated with the anti-angiogenic VEGF-A_165_b isoform, and VEGF-A_165_b appears to be upregulated in conditions with impaired limb revascularization [24]. The proposed mechanism involves the VEGF-A_165_b inhibition of VEGFR1 signaling, leading to both an inflammatory macrophage phenotype and impaired ischemic muscle vascularization [20]. However, other studies have identified the reduced expression of VEGF-A_165_b in patients with critical limb ischemia, suggesting our understanding of mechanisms governing splice variant signals involved in vascular remodeling and angiogenesis is somewhat incomplete [23,24,25]. Studies involving anti-VEGF therapy based on VEGF competitors or the blockade of VEGF receptors (VEGFR1 and VEGFR2) have not been specific to the individual splice variants, potentially limiting the true impact, given the opposing effects of VEGF-A_165_a and VEGF-A_165_b [56,57,58,59,60]. Of note, we previously found that macrophage *IL-1β*-deletion led to reduced VEGF-A_165_a but increased VEGF-A_165_b mRNA transcription, driven by IL-1β-dependent STAT3 and NF-κB activity [14]. Hence, the transcriptional modulation of VEGF-A exon 8 splice-site selection may be possible to express VEGF-A_165_a or VEGF-A_165_b for a respective desired effect. What the field currently needs is a model of isoform-specific, conditional *VEGF-A*-deletions to fully study the respective contributions of each isoform to the vascular remodeling phenotypes.

Previous literature highlighted the role of estradiol signaling in angiogenesis and VEGF-A signaling [61,62,63]; however, we did not identify any obvious differences in blood flow recovery when we assessed sex as a biological variable. This may reflect differences in the organ systems studied, the models of postdevelopmental angiogenesis being used, or cell-specific differences between endothelial and macrophage responses. It may be simply that our study is underpowered to detect subtle changes in macrophage responses between sexes. Future studies investigating the role of estradiol signaling in macrophages may require increased animal numbers and controls involving estrous cycle or gonadectomized animals to obtain a clearer assessment of the role of sex and hormonal signaling in regards to inflammatory angiogenesis and arteriogenesis.

Finally, one additional caveat to consider is that while an established and standardized PAD model, the femoral artery ligation model is an acute vascular injury model that is often carried out in the context of non-diseased vessels, as we did here. As our understanding of inflammatory angiogenesis and arteriogenesis improves, it will be important to assess these pathways in the context of atherosclerotic peripheral arteries, as the cellular responsiveness to proangiogenic signals may be altered or impaired by the disease process or disease risk factors like aging, diabetes mellitus, hyperlipidemia, hypertension, and tobacco smoking.

## 5. Conclusions

In summary, we have validated that early infiltrating macrophages are a major source of VEGF-A expression in ischemic muscle tissue within the first few days after vascular injury, using an experimental PAD model of hind limb ischemia. Further, macrophage VEGF-A expression, which we previously demonstrated to be transcriptionally regulated by IL-1β, is required for endothelial recruitment, leading to angiogenesis and arteriogenesis that improves blood flow recovery in the context of vascular injury. Understanding these mechanisms of macrophage-driven, postdevelopmental inflammatory angiogenesis and arteriogenesis will help identify cell-based therapeutic angiogenesis strategies that can promote an angiogenic state in diseased tissue, improving revascularization in pathophysiological conditions like PAD.

## Data Availability

The original contributions presented in this study are included in the article and Supplementary Material. Further inquiries can be directed to the corresponding author.

## Supplementary Materials

The following supporting information can be downloaded at: https://www.mdpi.com/article/10.3390/biomedicines13040828/s1, Figure S1: Clodronate liposome treated mice demonstrate reduced absolute numbers of circulating CD115^+^ cells. Related to Figure 1. Representative dot plots demonstrating gating for quantitation of CD45^+^CD3^+^, CD45^+^LY6G^+^, and CD45^+^CD115^+^ cell populations from peripheral blood of mice treated with control or clodronate liposomes; Figure S2: Control and clodronate liposome treated mice do not demonstrate significant sex-specific differences in blood flow recovery after hind limb ischemia. Related to Figure 1. Quantitative analysis from laser Doppler blood flow imaging of both control or clodronate liposome treated mice at indicated time points before and after femoral artery ligation (comparison between sex by ANOVA; n = 6 males or 6 females in each group) Data, mean ± SD; Figure S3: Control and mVEGF-A KO mice do not demonstrate significant sex-specific differences in blood flow recovery after hind limb ischemia. Related to Figure 3. Quantitative analysis from laser Doppler blood flow imaging of both control or mVEGF-A KO mice at indicated time points before and after femoral artery ligation (comparison between sex by ANOVA; n = 6 males or 6 females in each group). Data, mean ± SD; Figure S4: Transplant of LPS+INFγ-treated BMDMs from either wildtype (Control Mϕ) or VEGF-A-deleted (mVEGF-A KO Mϕ) mice into mVEGF-A KO does not demonstrate significant sex-specific differences in blood flow recovery after hind limb ischemia. Related to Figure 5. Quantitative analysis from laser Doppler blood flow imaging of both Control Mϕ or mVEGF-A KO Mϕ of each sex at indicated time points before and after femoral artery ligation (comparison between sex by ANOVA; n = 4 males or 4 females in each group). Data, mean ± SD.
