# Supplementary material for: Macrophage Proangiogenic VEGF-A Is Required for Inflammatory Arteriogenesis During Vascular Injury
Source: Biomedicines. 2025 Mar 31;13(4):828. doi: 10.3390/biomedicines13040828 (PMC12024885; doi:10.3390/biomedicines13040828)
Supplement: Supplementary file 1 [file biomedicines-13-00828-s001.zip › biomedicines-3492724-supplementary.pdf]

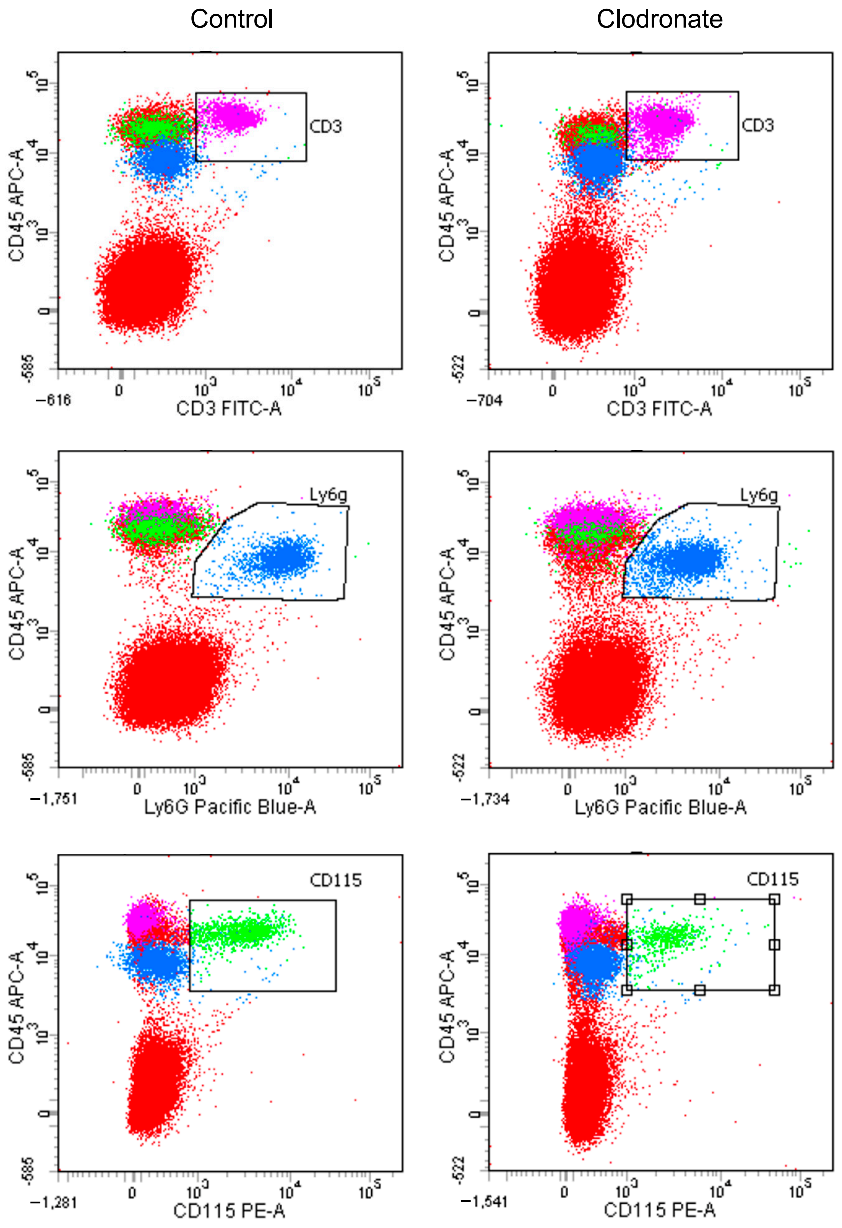

Supplemental Figure S1. Clodronate liposome treated mice demonstrate reduced absolute numbers of circulating CD115$^+$ cells. Related to Figure 1. Representative dot plots demonstrating gating for quantitation of CD45$^+$CD3$^+$, CD45$^+$LY6G$^+$, and CD45$^+$CD115$^+$ cell populations from peripheral blood of mice treated with control or clodronate liposomes.

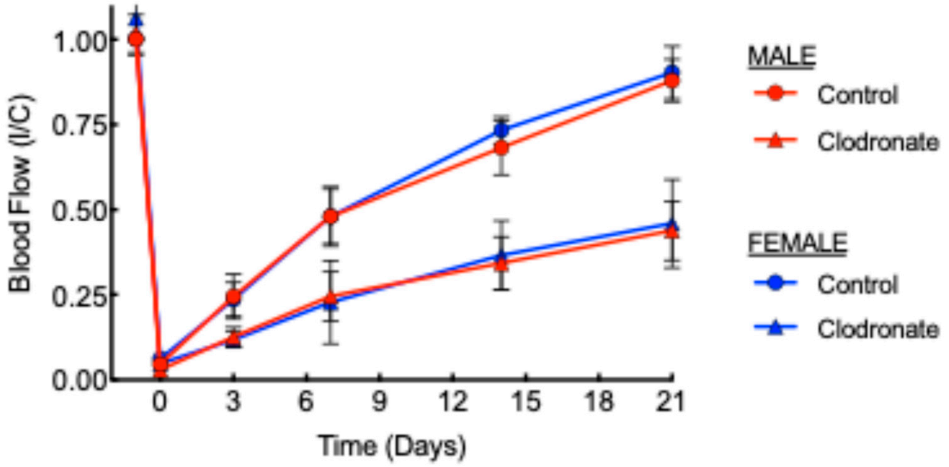

Supplemental Figure S2. Control and clodronate liposome treated mice do not demonstrate significant sex-specific differences in blood flow recovery after hind limb ischemia. Related to Figure 1. Quantitative analysis from laser Doppler blood flow imaging of both control or clodronate liposome treated mice at indicated time points before and after femoral artery ligation (comparison between sex by ANOVA; *n*=6 males or 6 females in each group). Data, mean ± SD.

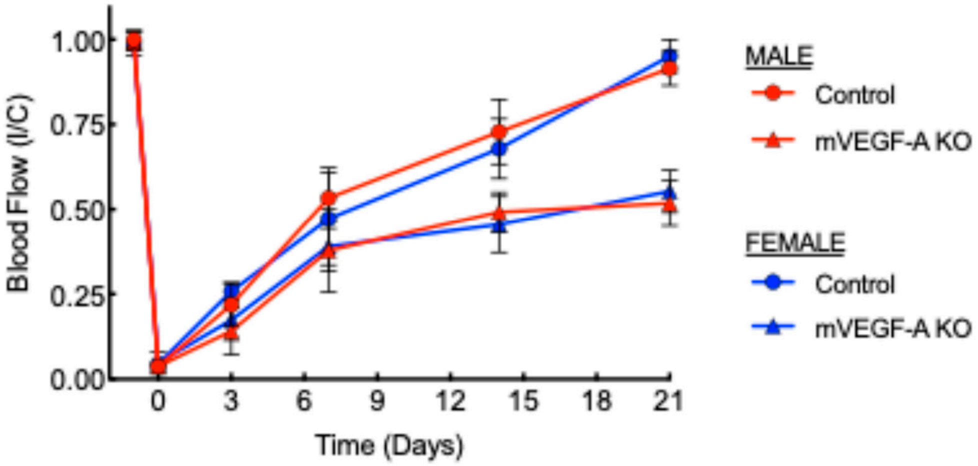

Supplemental Figure S3. Control and mVEGF-A KO mice do not demonstrate significant sex-specific differences in blood flow recovery after hind limb ischemia. Related to Figure 3. Quantitative analysis from laser Doppler blood flow imaging of both control or mVEGF-A KO mice at indicated time points before and after femoral artery ligation (comparison between sex by ANOVA; *n*=6 males or 6 females in each group). Data, mean ± SD.

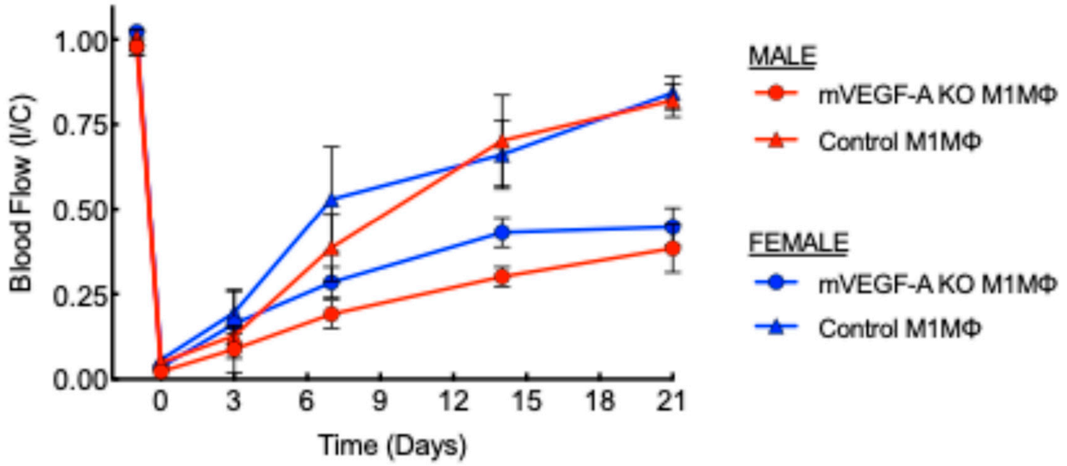

Supplemental Figure S4. Transplant of LPS+INFγ-treated BMDMs from either wild-type (Control Mϕ) or *VEGF-A*-deleted (mVEGF-A KO Mϕ) mice into mVEGF-A KO does not demonstrate significant sex-specific differences in blood flow recovery after hind limb ischemia. Related to Figure 5. Quantitative analysis from laser Doppler blood flow imaging of both Control Mϕ or mVEGF-A KO Mϕ of each sex at indicated time points before and after femoral artery ligation (comparison between sex by ANOVA; *n*=4 males or 4 females in each group). Data, mean ± SD.
